# Comparison of Intravenous versus Topical Tranexamic Acid in Nondeformity Spine Surgery: A Meta-Analysis

**DOI:** 10.1155/2020/7403034

**Published:** 2020-03-09

**Authors:** Zhencheng Xiong, Junyuan Liu, Ping Yi, Hao Wang, Mingsheng Tan

**Affiliations:** ^1^Graduate School of Peking Union Medical College, Chinese Academy of Medical Sciences, Beijing 100029, China; ^2^Department of Spine Surgery, China-Japan Friendship Hospital, Beijing 100029, China; ^3^Peking University Aerospace School of Clinical Medicine, Beijing 100029, China; ^4^Beijing University of Chinese Medicine, Beijing 100029, China

## Abstract

**Objective:**

Tranexamic acid (TXA), an antifibrinolytic agent, interferes with fibrinolysis and has been used for many years to reduce blood loss during spine surgery. The purpose of our meta-analysis was to compare the effect of intravenous versus topical administration of TXA in patients undergoing nondeformity spine surgery.

**Methods:**

We searched multiple databases, including PubMed, Embase, the Cochrane library, CNKI, WanFang database, and VIP to find studies that met the inclusion criteria. A meta-analysis was performed according to the guidelines of the Cochrane Reviewer's Handbook.

**Results:**

Eight randomized controlled trials (RCTs) were identified, including 660 patients. The surgical methods used in the included studies were nondeformity spine surgery. No significant differences were found in the two groups regarding total blood loss, intraoperative blood loss, hidden blood loss, hematocrit, hemoglobin, fibrinogen, postoperative prothrombin time (PT), postoperative activated partial thromboplastin time (APTT), drainage volume, and blood transfusion rate. There were statistically significant differences in the two groups in terms of preoperative PT (MD = −0.39, 95% CI: [−0.63, −0.15], *P*=0.002) and preoperative APTT (MD = 1.12, 95% CI: [0.57, 1.68], *P*=0.002) and preoperative APTT (MD = 1.12, 95% CI: [0.57, 1.68],

**Conclusion:**

During nondeformity spine surgery, intravenous administration of TXA did not have a significant effect on the decrease of blood loss and blood transfusion rate compared with the topical group. According to the pooled analysis of PT and APTT, intravenous and topical application of TXA may have different effects on the coagulation pathway. More high-quality RCTs are needed to explore the optimal dosage, method, timing in the future in order to recommend TXA widespread use in spine surgery.

## 1. Introduction

Spine surgery is usually accompanied by significant blood loss during the perioperative period, which may lead to acute anemia and even serious complications [1]. These conditions inevitably require significant blood transfusions and carry additional risks, such as infectious disease transmission, hemolysis, postoperative spinal epidural hematoma formation, anaphylactic reactions, and economic burden [2]. At present, there are many interventions applied to clinical practice, which are mainly divided into two types. One is to supplement blood loss in a variety of ways, and the other is to stop bleeding with drugs or materials. Among these hemostatic drugs, tranexamic acid (TXA) interferes with fibrinolysis and has been used for many years to reduce blood loss during spine surgery [3].

TXA, an antifibrinolytic agent, reversibly and competitively binds to lysine-binding domains on plasminogen, plasmin, and tissue plasminogen activator [4]. There have been many studies demonstrating that intravenous or topical administration of TXA could reduce blood loss and allogenic blood transfusions without a high risk of complications such as deep vein thrombosis (DVT), pulmonary embolism (PE), or wound infection [4, 5]. Recently, some studies have reported a comparison of the efficacy and safety of intravenous versus topical administration of TXA during spine surgery [6–13]. However, the optimal administration route of TXA remains controversial during spine surgery. Therefore, we conducted this meta-analysis to evaluate the efficacy and safety of intravenous versus topical administration of TXA during nondeformity spine surgery from randomized controlled trials (RCTs).

## 2. Materials and Methods

We conducted this meta-analysis based on the Preferred Reporting Items for Systematic Reviews and Meta-Analyses (PRISMA) statement [14].

### 2.1. Search Strategy

First of all, to obtain all the literature relevant to our research, two researchers independently searched multiple databases using keywords combined with free words according to Cochrane Collaboration guidelines, such as PubMed (1966 to August 1, 2019), Embase (1980 to August 1, 2019), the Cochrane library (1966 to August 1, 2019), China National Knowledge Infrastructure databases (CNKI) (1980 to August 1, 2019), WanFang database (1990 to August 1, 2019), and Chinese Scientific Journal Database (VIP) (1990 to August 1, 2019). Using Medical Subject Headings (MeSH) terms and corresponding keywords, we searched for the following terms “tranexamic acid,” “TXA,” “intravenous,” “topical,” “blood loss,” and “spine surgery” with the Boolean operators “AND or OR.” Potentially related literature was also searched from the reference lists in all included studies. The above-retrieved literature was screened by two researchers independently reading the titles and abstracts. As a final step, we further filtered the selected literature by reading the full text. All disagreeable literature was resolved after the discussion.

### 2.2. Study Selection

All studies included in this meta-analysis met the following criteria: (1) All studies involved the comparison of the effect of intravenous versus topical administration of TXA in patients undergoing nondeformity spine surgery; (2) The full text of the included literature could be obtained, and the outcome measures of the effect of intravenous versus topical administration of TXA, total blood loss (TBL), intraoperative blood loss (IBL), hidden blood loss (HBL), blood transfusion rate, hematocrit (HCT), prothrombin time (PT), fibrinogen (FIB), activated partial thromboplastin time (APTT), hemoglobin (Hb), and drainage volume could be extracted.

This meta-analysis excluded the following studies: (1) The patients had received other strategies to prevent blood loss; (2) the patients with a history of thromboembolic events (DVT or PE), clotting disorders, and severe cardiovascular dysfunction; (3) studies were not suitable with the inclusive criteria.

### 2.3. Data Extraction and Quality Assessment

Data were independently extracted by two researchers. Then another researcher collected the data using a spreadsheet. After discussion, the disagreements in the process of data extraction were solved. The following data were extracted: first author, year of publication, country, study type, number of participants (intravenous: topical), surgical methods, age, gender, body mass index (BMI), anesthesia methods, intervention (intravenous: topical), and transfusion criteria.

Two researchers conducted a quality assessment of each included RCT based on the Cochrane Handbook for Systematic Reviews [15]. We created a “risk of bias” table that included the following elements: (1) Random sequence generation; (2) allocation concealment; (3) blinding of participant and personnel; (4) blinding of outcome assessment; (5) incomplete outcome data; (6) selective reporting; (7) other bias. Every section had a high risk of bias, low risk of bias, and unclear risk of bias depending on the actual content of the included study [15].

### 2.4. Statistical Analysis

Different studies had compared intravenous versus topical groups according to TBL, IBL, HBL, blood transfusion rate, HCT, PT, APTT, FIB, Hb, and drainage volume. We aggregated and calculated data for the same outcome measure in all studies and placed them in the same table. We divided some outcome measures into subgroups based on the classification or recording time. The dichotomous data was analyzed by using risk ratio (RR) and their 95% confidence interval (CI), such as blood transfusion rate. The continuous data was analyzed by using weighted mean differences (WMD) and their 95% CI, such as TBL, IBL, HBL, HCT, PT, APTT, FIB, Hb, and drainage volume. We calculated the statistical heterogeneity using a Chi-squared test and *I*^2^ test. When the values of *I*^2^ are 25%, 50%, and 75%, they are considered as low, medium, and high heterogeneity, respectively [16]. When *I*^2^ > 50%, *P* < 0.1, we performed a random-effect model; otherwise, a fixed-effect model was performed. We performed this meta-analysis using RevMan 5.3 for Windows (Cochrane Collaboration, Oxford, UK). The results of this meta-analysis were considered statistically significant if *P* < 0.05.

## 3. Results

### 3.1. Selection of Studies

**Figure 1:**
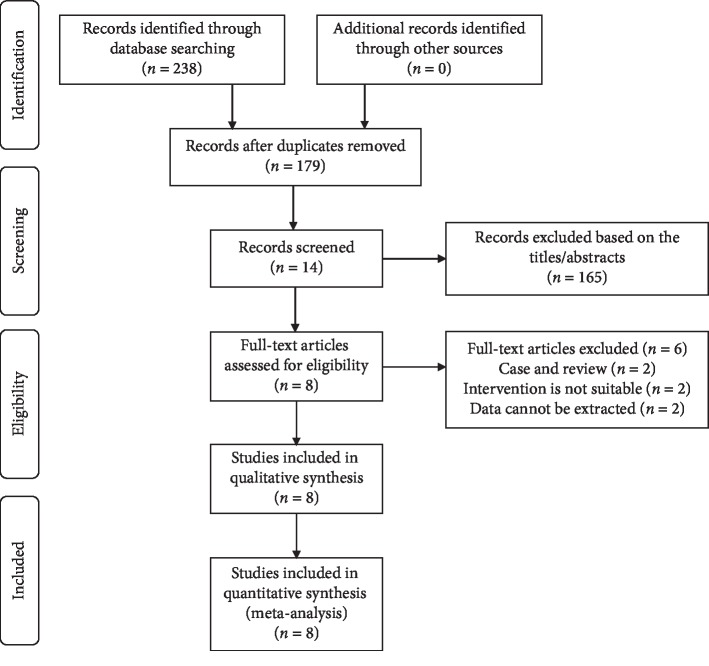
Flow diagram of the study selection process for the meta-analysis.

Firstly, we used keywords and free words to search in multiple databases, and finally confirmed 238 records. Then, a total of 14 records were screened out by reading the titles and abstracts to remove duplicate and irrelevant records. We excluded letter or review, and records for which data could not be extracted according to the inclusion criteria. Finally, eight RCTs [6–13] met the criteria for data extraction and meta-analysis.  Figure 1 shows the search strategy and selection process [6–13].

### 3.2. Study Characteristics

A total of 8 RCTs published between 2016 and 2019 were included in this meta-analysis. Characteristics of all studies are shown in Table 1. All studies compared the effect of intravenous versus topical administration of TXA in patients undergoing nondeformity spine surgery. The number of patients in the intravenous group (333 patients) was higher than in the topical group (327 patients) [6–13]. The surgical method used in four studies [6, 7, 9, 11] was posterior lumbar decompression and fusion. These studies include a total of five surgical methods, all of which were nondeformity spine surgery [6–13]. In the included studies, Wang et al. [8] claimed that in the intravenous group, intravenous drip of TXA at a dose of 15 mg/kg was given half an hour before the operation. In the topical group, intraoperative topical application of 3 g TXA was prescribed before wound closure [8]. Mu et al. [7] claimed that in the intravenous group, intravenous drip of TXA at a loading dose of 15 mg/kg was given half an hour before the operation, and at a maintenance dose of 1 mg/kg during surgery. In the topical group, topical application of 1 g TXA was prescribed before wound closure [7]. In three studies, Hb level below 70 g/L was considered indications for blood transfusion [6, 7, 11], while in one study was below 80 g/L [12].

### 3.3. Risk of Bias

**Figure 2:**
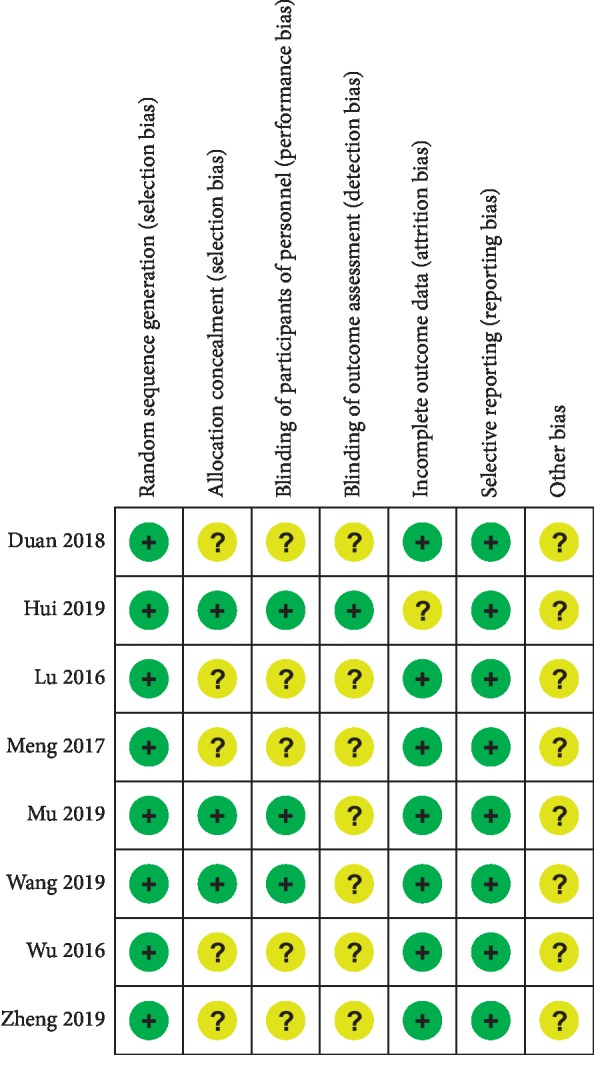
Risk of bias summary: +, low risk of bias; −, high risk of bias; ?, bias unclear.

A total of 3 RCTs were considered to have a low risk of bias [7–9]. Random sequence generation was found in eight studies [6–13]. Allocation concealment and blinding of participants and personnel were found in 3 studies [7–9]. Blinding of outcome assessment was found in one study [9]. As shown in Figure 2, none of the eight RCTs found selective reports [6–13].

### 3.4. Outcomes of the Meta-Analysis

We summarized the evaluation tools to compare the effect of intravenous versus topical administration of TXA during nondeformity spine surgery after carefully reading and analyzing the included articles, including blood loss (TBL, IBL, and HBL), blood transfusion rate, HCT, PT, APTT, FIB, Hb, and drainage volume [6–13]. Among them, blood loss is the primary outcome measure. Results of the meta-analysis of outcome measures are shown in Table 2.

#### 3.4.1. Blood Loss

**Figure 3:**
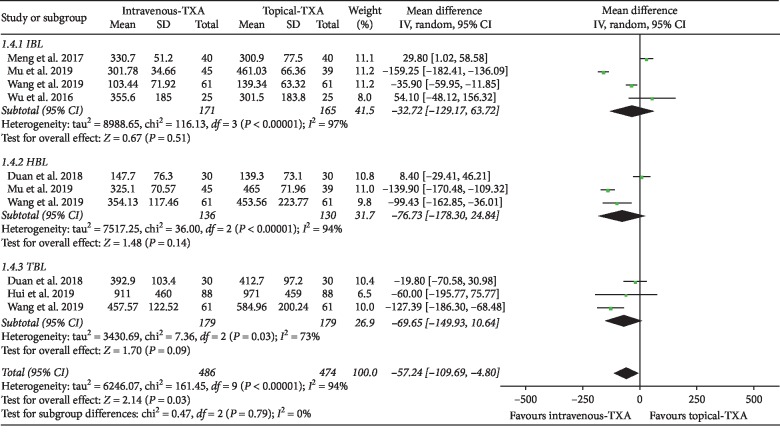
Forest plot showing the effect of intravenous administration of TXA on blood loss compared with the topical group during nondeformity spine surgery (TBL: total blood loss; IBL: intraoperative blood loss; HBL: hidden blood loss).

As shown in Figure 3, the forest plot shows the effect of intravenous administration of TXA on blood loss compared with the topical group during nondeformity spine surgery. Six RCTs used blood loss as the primary outcome measurement [6–9, 11, 12]. Blood loss was divided into 3 subgroups according to different types. A total of 4 studies (336 patients) [6–8, 11] provided data on IBL, 3 studies (266 patients) [7, 8, 12] provided data on HBL, and 3 studies (358 patients) [8, 9, 12] provided data on TBL. A random-effect model was used because significant heterogeneity was found among the studies (*I*^2^ > 50%, *P* < 0.1). There were no statistically significant differences on IBL, HBL, and TBL between the two groups based on the results of the pooled analysis (IBL: MD = −32.72, 95% CI: [−129.17, 63.72], *P* = 0.51, *I*^2^ = 97%; HBL: MD = −76.73, 95% CI: [−178.30, 24.84], *P* = 0.14, *I*^2^ = 94%; TBL: MD = −69.65, 95% CI: [−149.93, 10.64], *P* = 0.09, *I*^2^ = 73%).

#### 3.4.2. Hb Level and HCT Level

**Figure 4:**
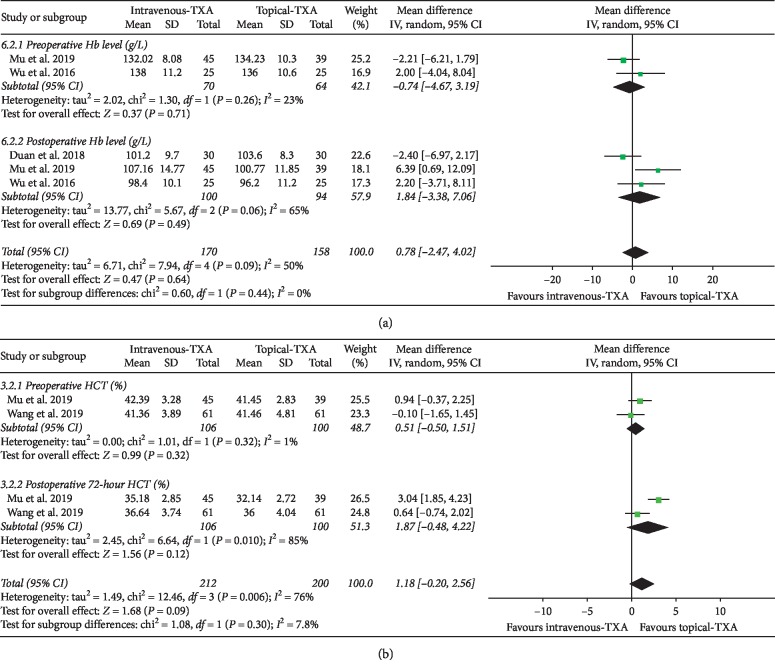
(a) Forest plot showing the effect of intravenous administration of TXA on Hb level compared with the topical group during nondeformity spine surgery (Hb: hemoglobin). (b) Forest plot showing the effect of intravenous administration of TXA on HCT compared with the topical group during nondeformity spine surgery (HCT: hematocrit).

As shown in Figure 4(a), the forest plot shows the effect of intravenous administration of TXA on Hb level compared with the topical group during nondeformity spine surgery. Hb was divided into 2 subgroups according to different time points. A total of 2 studies (134 patients) [6, 7] provided data on preoperative Hb, and 3 studies (194 patients) [6, 7, 12] provided data on postoperative Hb. A random-effect model was used because significant heterogeneity was found among the studies (*I*^2^ > 50%, *P* < 0.1). There were no statistically significant differences on Hb level between the two groups based on the results of the pooled analysis (preoperative Hb: MD = −0.74, 95% CI: [−4.67, 3.19], *P* = 0.71, *I*^2^ = 23%; postoperative Hb: MD = 1.84, 95% CI: [−3.38, 7.06], *P* = 0.49, *I*^2^ = 65%).

As shown in Figure 4(b), the forest plot shows the effect of intravenous administration of TXA on HCT compared with the topical group during nondeformity spine surgery. HCT was divided into 2 subgroups according to different time points. A total of 2 studies (206 patients) provided data on preoperative HCT, and 2 studies (206 patients) provided data on postoperative HCT [7, 8]. A random-effect model was used because significant heterogeneity was found among the studies (*I*^2^ > 50%, *P* < 0.1). There were no statistically significant differences on HCT level between the two groups based on the results of the pooled analysis (preoperative HCT: MD = 0.51, 95% CI: [−0.50, 1.51], *P* = 0.32, *I*^2^ = 1%; postoperative HCT: MD = 1.87, 95% CI: [−0.48, 4.22], *P* = 0.12, *I*^2^ = 85%).

#### 3.4.3. PT and APTT

**Figure 5:**
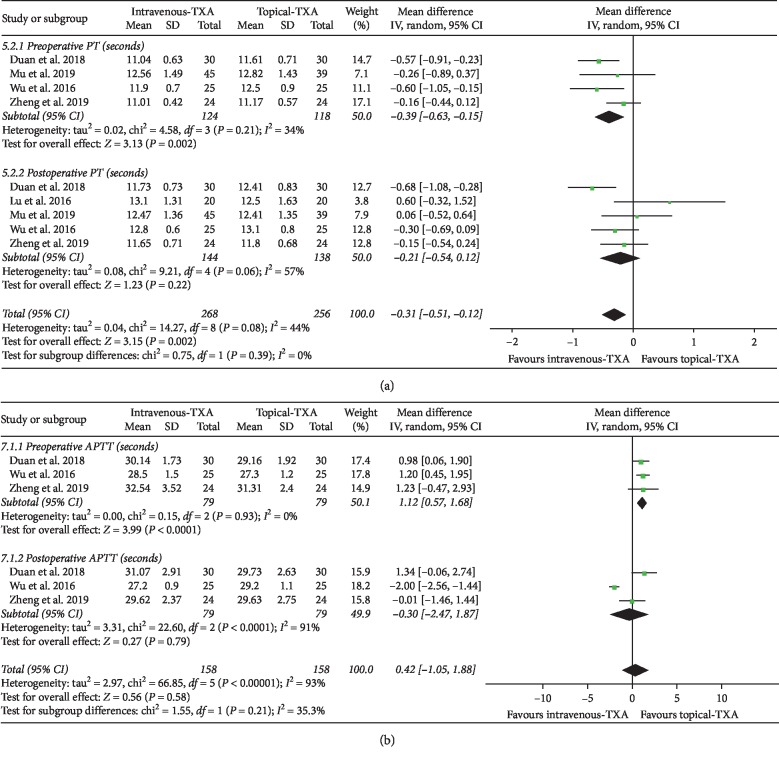
(a) Forest plot showing the effect of intravenous administration of TXA on PT compared with the topical group during nondeformity spine surgery (PT: prothrombin time). (b) Forest plot showing the effect of intravenous administration of TXA on APTT compared with the topical group during nondeformity spine surgery (APTT: activated partial thromboplastin time).

As shown in Figure 5(a), the forest plot shows the effect of intravenous administration of TXA on PT compared with the topical group during nondeformity spine surgery. PT was divided into 2 subgroups according to different time points. A total of 4 studies (242 patients) [6, 7, 12, 13] provided data on preoperative PT, and 5 studies (282 patients) [6, 7, 10, 12, 13] provided data on postoperative PT. A random-effect model was used because significant heterogeneity was found among the studies (*I*^2^ > 50%, *P* < 0.1). Based on the results of the pooled analysis, there was a statistically significant difference on preoperative PT between the two groups (MD = −0.39, 95% CI: [−0.63, −0.15], *P* = 0.002, *I*^2^ = 34%). However, there were no statistically significant differences on postoperative PT between the two groups based on the results of the pooled analysis (MD = −0.21, 95% CI: [−0.54, 0.12], *P* = 0.22, *I*^2^ = 57%).

As shown in Figure 5(b), the forest plot shows the effect of intravenous administration of TXA on APTT compared with the topical group during nondeformity spine surgery. APTT was divided into 2 subgroups according to different time points. A total of 3 studies (158 patients) provided data on preoperative APTT, and 3 studies (158 patients) provided data on postoperative APTT [6, 12, 13]. A random-effect model was used because significant heterogeneity was found among the studies (*I*^2^ > 50%, *P* < 0.1). There was a statistically significant difference on preoperative APTT between the two groups (MD = 1.12, 95% CI: [0.57, 1.68], *P* < 0.0001, *I*^2^ = 0%). However, there were no statistically significant differences on postoperative APTT between the two groups based on the results of the pooled analysis (MD = −0.30, 95% CI: [−2.47, 1.87], *P* = 0.79, *I*^2^ = 91%).

#### 3.4.4. FIB Level

**Figure 6:**
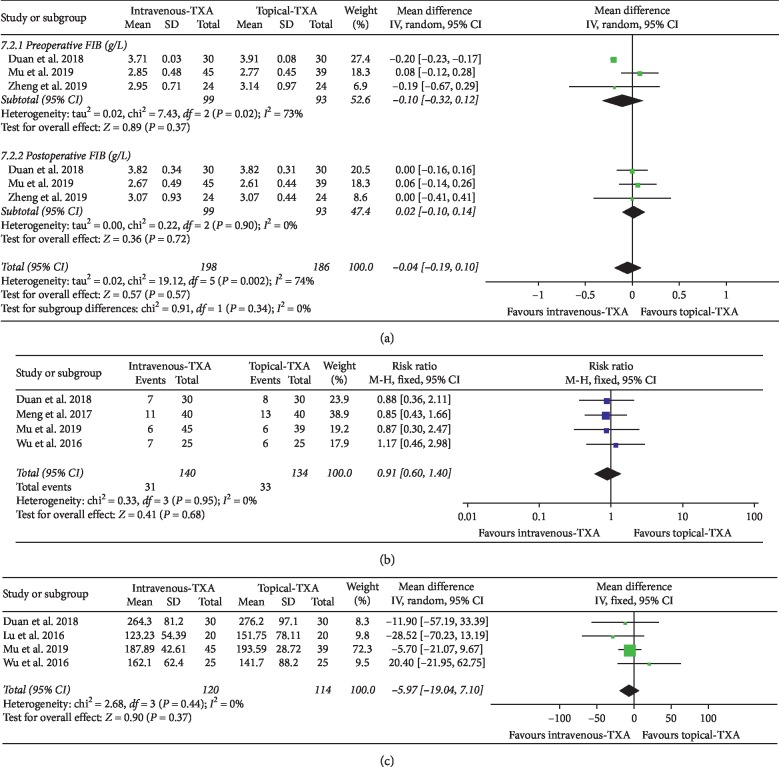
(a) Forest plot showing the effect of intravenous administration of TXA on FIB compared with the topical group during nondeformity spine surgery (FIB: fibrinogen). (b) Forest plot showing the effect of intravenous administration of TXA on blood transfusion rate compared with the topical group during nondeformity spine surgery (RR: risk ratio). (c) Forest plot showing the effect of intravenous administration of TXA on drainage volume compared with the topical group during nondeformity spine surgery.

As shown in Figure 6(a), the forest plot shows the effect of intravenous administration of TXA on FIB level compared with the topical group during nondeformity spine surgery. FIB was divided into 2 subgroups according to different time points. A total of 3 studies (192 patients) provided data on preoperative FIB, and 3 studies (192 patients) provided data on postoperative FIB [7, 12, 13]. A random-effect model was used because significant heterogeneity was found among the studies (*I*^2^ > 50%, *P* < 0.1). There were no statistically significant differences on FIB level between the two groups based on the results of the pooled analysis (preoperative FIB: MD = −0.10, 95% CI: [−0.32, 0.12], *P* = 0.37, *I*^2^ = 73%; postoperative FIB: MD = 0.02, 95% CI: [−0.10, 0.14], *P* = 0.72, *I*^2^ = 0%).

#### 3.4.5. Blood Transfusion Rate

As shown in Figure 6(b), the forest plot shows the effect of intravenous administration of TXA on blood transfusion rate compared with the topical group during nondeformity spine surgery. A total of 4 studies (274 patients) provided data on blood transfusion rate [6, 7, 11, 12]. A fixed-effect model was used because significant heterogeneity was found among the studies (*I*^2^ < 50%). There were no statistically significant differences on blood transfusion rate between the two groups based on the results of the pooled analysis (RR = 0.91, 95% CI: [0.60, 1.40], *P* = 0.68, *I*^2^ = 0%).

#### 3.4.6. Drainage Volume

As shown in Figure 6(c), the forest plot shows the effect of intravenous administration of TXA on drainage volume compared with the topical group during nondeformity spine surgery. A total of 4 studies (234 patients) provided data on drainage volume [6, 7, 10, 12]. A fixed-effect model was used because significant heterogeneity was found among the studies (*I*^2^ < 50%). There were no statistically significant differences on drainage volume between the two groups based on the results of the pooled analysis (MD = −5.97, 95% CI: [−19.04, 7.10], *P* = 0.37, *I*^2^ = 0%).

#### 3.4.7. Adverse Events

In one study, a total of 3 patients were found to have postoperative wound infection (intravenous group: 2, topical group: 1) [7]. One RCT stated that a total of 7 patients developed different complications including cerebrospinal fluid leakage, stress ulcers, lung infections, and urinary tract infections (intravenous group: 4, topical group: 3) [11]. A total of 6 studies (404 patients) claimed that no DVT and PE were found at the final follow-up evaluations [6–8, 10, 12, 13]. Considering the relatively small number of studies and patients, more high-quality studies are needed to compare adverse events in both groups.

### 3.5. Publication Bias

**Figure 7:**
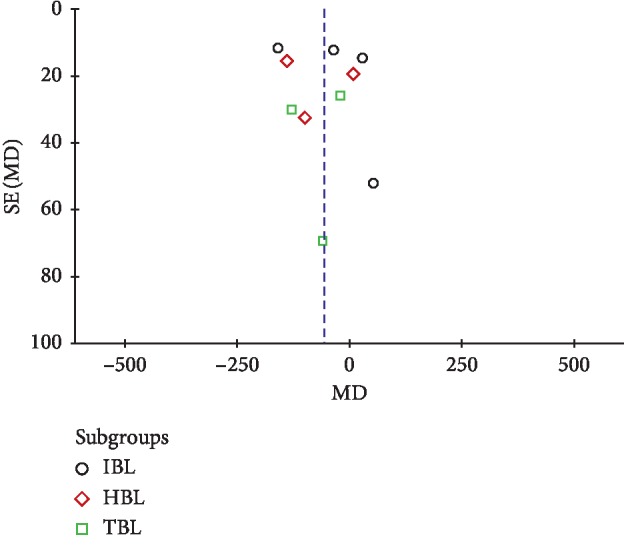
Funnel plot to detect publication bias for studies comparing blood loss between two groups during nondeformity spine surgery.

Funnel plot was usually used to assess publication bias and was usually only performed in at least 10 studies. The number of studies included will have an effect on the effectiveness of the funnel plot to test publication bias. If too few studies are included, the funnel plot's testing power will decrease accordingly. As shown in Figure 7, we used funnel plots to detect publication bias for studies comparing blood loss between two groups during nondeformity spine surgery. Visual inspection of the funnel plots showed asymmetry. The asymmetry of the funnel plots may be due to insufficient trials and statistical heterogeneity.

### 3.6. Sensitivity Analysis

When *I*^2^ > 50%, this means that the included studies are highly heterogeneous. If necessary, a sensitivity analysis was performed to identify the origin of the significant heterogeneity. The heterogeneity of blood loss, postoperative Hb, postoperative APTT, postoperative PT, postoperative HCT, and preoperative FIB were greater than 50%. Therefore, we performed sensitivity analysis separately to assess the reliability of the results. Through careful analysis of the included studies, we found that the following reasons may be the source of heterogeneity: (1) The patients included in each article have different diseases, undergoing different spine surgeries, different doses of TXA; (2) limited research meet the inclusion criteria; (3) the collection time of the data was not exactly the same. We removed the literature one by one and found that the heterogeneity results were still greater than 50%. Therefore, more high-quality RCTs are needed in the future to determine the source of heterogeneity.

## 4. Discussion

TXA, an antifibrinolytic drug, was primarily used to treat or prevent excessive blood loss in orthopedic, cardiac, and spine surgery [1]. TXA can be administered in a variety of ways, including intravenous, topically, orally, or a combination thereof [1]. TXA is usually given intravenously as a bolus prior to the incision and then maintained as a continuous infusion throughout the procedure [4]. A large number of prospective studies and meta-analysis had shown that both intravenous and topical TXA could reduce blood loss and blood transfusion requirements in comparison to an equal volume of 0.9% saline in orthopedic surgery [4, 5]. Zhang et al. [17] in 2019 conducted a meta-analysis of 11 studies, a total of 748 patients, and were able to show that intravenous TXA could effectively reduce IBL and perioperative blood transfusion during multilevel spine surgery, and could restore Hb level after surgery. Elmose et al. [18] demonstrated that there was no statistically significant effect of intravenous TXA on intraoperative blood loss, operative time, or complications during minor lumbar spine surgery. However, intravenous TXA may have potentially adverse effects on patients, including DVT, PE, and myocardial infarction [18, 19]. Intravenous TXA is usually avoided in patients with a previous history of myocardial infarction, DVT/ PE, stroke, and seizure disorders [20–23]. In addition, some research teams had published different conclusions. Ko et al. [24] showed that there was no significant difference in the incidence of DVT between intravenous TXA and placebo. Studies had shown that intravenous TXA could effectively reduce blood loss without increasing the risk of thrombotic events during spine deformity surgery [25].

The potential thrombotic risk of intravenous application of TXA promoted the topical administration of TXA as a potentially safer and more targeted intraoperative hemostasis strategy [2]. Topical TXA has been widely used to reduce blood loss in orthopedic, cardiac, and thoracic surgery [26]. Topical TXA can provide direct and local high concentration drugs at the bleeding site and avoid the systemic exposure of TXA [1, 2, 27]. Sudrasert et al. [26] demonstrated that topical TXA could effectively decrease postoperative transfusion requirements and postoperative blood loss in patients undergoing long-segment instrumented posterior spinal fusion. A latest meta-analysis in 2018 showed that the topical administration of TXA in spine surgery decreased TBL and drainage volume and reserved higher postoperative Hb level without increasing the risk of DVT, PE, or wound infection [1]. Some studies had also shown that topical TXA was only used before the wound was closed, which did not help blood loss during the procedure [28]. Whether it is intravenous TXA or topical TXA, there are certain side effects. Severe systemic side effects after intravenous injection of TXA are rare but do exist. Some studies have also shown that the combined application is more effective than the single application [8]. However, the optimal route of TXA administration remains controversial. Because few studies have compared the efficiency and safety of intravenous versus topical administration of TXA during nondeformity spine surgery. Moreover, no meta-analysis has been conducted on the efficiency and safety of intravenous and topical use of TXA in nondeformity spine surgery. Therefore, we conducted this meta-analysis. In the future, more high-quality RCTs will be needed to complement existing conclusions. For different types of spinal diseases, find the best application.

According to the analysis of blood loss (TBL, IBL, and HBL), blood transfusion rate, HCT, PT, APTT, FIB, Hb level, and drainage volume, different results can be seen in different outcome measures. Previous systematic studies have shown that the intravenous administration of TXA was more effective than placebo in reducing IBL [3]. However, based on the data from the literature included in this meta-analysis, we found that there were no significant differences on IBL between the intravenous and topical administration of TXA. This indicated that both the intravenous and topical administration of TXA was more effective in reducing IBL during nondeformity spine surgery. Previous systematic studies have shown that there were no significant differences on HCT, FIB, drainage volume, and blood transfusion rate between the intravenous and topical administration of TXA in total hip or knee arthroplasty [4]. In our meta-analysis, the same outcome measures have similar results. This indicates that there were no significant differences on HCT, FIB, drainage volume, and blood transfusion rate between the intravenous and topical administration of TXA during nondeformity spine surgery. In one study, Xie et al. [4] demonstrated that intravenous administration of TXA was associated with significantly smaller maximum Hb drop in total hip arthroplasty. Based on the data from the literature included in our meta-analysis, we found that there was no significant difference between the two groups regarding Hb level.

PT is usually used in combination with APTT to measure the extrinsic and intrinsic pathways of coagulation [29]. PT is not only an important indicator to check the function of extrinsic coagulation system, but also an important monitoring indicator for clinical anticoagulation therapy [30]. APTT is the most commonly used sensitive screening test to reflect the coagulation activity of intrinsic coagulation system in clinic [28]. Through the pooled analysis of PT and APTT, we found that there were statistically significant differences before surgery, and there were no statistically significant differences after operation. The above results indicated that PT in the intravenous group changed more significantly than that in the topical group during nondeformity spine surgery. On the contrary, APTT in the topical group changed more significantly than that in the intravenous group. Intravenous and topical administration of TXA may have different effects on the coagulation pathway during nondeformity spine surgery. However, the inclusion of studies is relatively limited, and more high-quality studies are needed to reveal the mechanisms and effects of the two application approaches on coagulation pathways.

Among the included studies, various studies had used different protocols for intravenous and topical TXA [6–13]. As a result, the optimal dose for maximal effects of each delivery method is not compared directly in some studies. In one study, topical TXA was used in two doses, including 0.5% TXA and 1% TXA [13]. The results showed that 1% TXA was superior to 0.5% TXA in reducing intraoperative bleeding and drainage. Therefore, we chose to extract the relevant data of 1% TXA. In the other seven studies, both intravenous TXA and topical TXA were fixed doses and were not completely consistent [6–12]. The optimal dosage and application of TXA has been a hot topic in clinical discussion. In one study, the high-dose and low-dose groups received 10 and 5 mg/kg of bolus loading dose and 2 and 1 mg/kg of continuous infusion until 5 h after surgery [31]. They claimed better hemostatic effects in the high-dose group. In other studies, 10 mg has become a low-dose group, and whether it is still superior to higher doses remains controversial [32]. A meta-analysis showed that high-dose TXA as the optimal administration that had the best efficacy and safety [33]. However, the optimal dose of intravenous TXA and topical TXA in spine surgery is currently controversial. And the relationship between TXA dose and blood loss control is unclear. In addition, the limited number of studies meeting the inclusion criteria has resulted in a limitation that cannot directly compare the optimal doses for each method of administration [34].

### 4.1. Limitations

This is the first meta-analysis to compare the efficacy and safety of intravenous versus topical administration of TXA in nondeformity spine surgery. However, there had been limited studies comparing the effect of intravenous versus topical administration of TXA in spine surgery. Therefore, this article also has some limitations. Firstly, the number of included studies is quite limited, and many studies have incomplete data and relatively low quality. Secondly, various studies used different protocols for intravenous and topical TXA, thus the optimal dose for maximal effects of each delivery method is not compared directly. Thirdly, the administration time of intravenous TXA and topical TXA was not the same in different studies. Fourthly, most studies lacked the details of random sequence generation, allocation concealment, and blind implementation. Fifthly, all research teams belong to one country and the surgical methods are not uniform. Finally, the transfusion criteria were not uniform, and the evaluation time of the outcome measurement was also inconsistent.

## 5. Conclusion

There are many studies that have confirmed that both intravenous TXA and topical TXA can effectively reduce blood loss and blood transfusion requirements during spine surgery. However, there is no meta-analysis comparing the effects of intravenous TXA versus topical TXA in nondeformity spine surgery. The results of the above analysis indicated that there were no significant differences in the effect of intravenous administration of TXA on blood loss, Hb, HCT, FIB, postoperative PT and APTT, drainage volume, and blood transfusion rate compared with the topical group. There were statistically significant differences on preoperative PT and APTT between the two groups. Intravenous and topical application of TXA may have different effects on the function of the coagulation system during nondeformity spine surgery. Due to the limited number of studies, it is not sufficient to compare the incidence of adverse events between the two groups, and the generalizability of conclusions is relatively limited. Therefore, more high-quality RCTs are needed in spine surgery patients to determine the optimal TXA dosage and application method in the future to supplement the existing conclusions.

## Figures and Tables

**Table 1:** Characteristics of all the trials included in the meta-analysis.

Study	Country	Study type	Hemostatic agent	Surgical methods	Disease diagnosis	Anesthesia methods	Group	Age (years)	Gender M : F	BMI (kg/m^2^)	TXA dosing (loading + maintenance)	Transfusion criteria
Wang et al, [8]	China	RCT	TXA	Percutaneous pedicle screw fixation	Thoracolumbar fracture	General anesthesia	Intravenous topical	45.43 ± 8.18^†^ 45.72 ± 9.96^‡^	61 (34/27)^†^ 61 (37/24)^‡^	21.26 ± 2.26^†^ 21.37 ± 2.17^‡^	15 mg/kg (loading)^†^ 3 g before incision closure^‡^	NP^†^ NP^‡^
Mu et al, [7]	China	RCT	TXA	Posterior lumbar decompression and fusion	Lumbar degenerative disease	General anesthesia	Intravenous topical	54.20 ± 7.37^†^ 51.77 ± 8.13^‡^	45 (27/18)^†^ 39 (22/17)^‡^	24.83 ± 1.95^†^ 24.72 ± 1.82^‡^	15 mg/kg + 1 mg/kg/h^†^ 1 g before incision closure^‡^	Hb < 70 g/L^†^ Hb < 70 g/L^‡^
Hui et al, [9]	China	RCT	TXA	Lumbar decompression and fusion	Lumbar degenerative disease	General anesthesia	Intravenous topical	NP^†^ NP^‡^	NP^†^ NP^‡^	NP^†^ NP^‡^	10 mg/kg + 1 mg/kg/h^†^ 0.5 g before incision closure^‡^	NP^†^ NP^‡^
Wu et al, [6]	China	RCT	TXA	Posterior lumbar decompression and fusion	Lumbar degenerative disease	General anesthesia	Intravenous topical	NP^†^ NP^‡^	NP^†^ NP^‡^	NP^†^ NP^‡^	100 ml (loading)^†^ 20 ml before incision closure^‡^	Hb ≤ 70 g/L^†^ Hb ≤ 70 g/L^‡^
Lu et al, [10]	China	RCT	TXA	One stage posterior surgery of thoracic spinal tuberculosis, interbody fusion and internal fixation	Thoracic spinal tuberculosis	General anesthesia	Intravenous topical	NP^†^ NP^‡^	NP^†^ NP^‡^	NP^†^ NP^‡^	10 mg/kg (loading)^†^ 10 mg/kg^‡^	NP^†^ NP^‡^
Meng et al, [11]	China	RCT	TXA	Posterior lumbar decompression and fusion	Lumbar degenerative disease	General anesthesia	Intravenous topical	62.30 ± 5.40^†^ 61.10 ± 5.80^‡^	40 (23/17)^†^ 40 (24/16)^‡^	25.20 ± 5.30^†^ 27.50 ± 4.70^‡^	15 mg/kg (loading)^†^ NP^‡^	Hb ≤ 70 g/L^†^ Hb ≤ 70 g/L^‡^
Duan et al, [12]	China	RCT	TXA	Posterior cervical single-door laminoplasty	Cervical spondylitis myelopathy	General anesthesia	Intravenous topical	57.00 ± 4.11^†^ 57.10 ± 4.82^‡^	30 (17/13)^†^ 30 (15/15)^‡^	NP^†^ NP^‡^	10 mg/kg (loading)^†^ 1 g before incision closure^‡^	Hb ≤ 80 g/L^†^ Hb ≤ 80 g/L^‡^
Zheng et al, [13]	China	RCT	TXA	Multisegment, thoracolumbar posterior bone graft fusion and internal fixation	Lumbar degenerative disease	General anesthesia	Intravenous topical	NP^†^ NP^‡^	NP^†^ NP^‡^	NP^†^ NP^‡^	100 ml (loading)^†^ 100 ml before incision closure^‡^	NP^†^ NP^‡^

^†^Intravenous group; ^‡^Topical group. RCT, randomized controlled trial; TXA, tranexamic acid; M, male; F, female; BMI, body mass index; NP, not provided.

**Table 2:** Results of the meta-analysis of outcome measures.

Outcome and Subgroup	Number of studies	Patients I: T	RR/MD (95% CI)	*p* Value	Heterogeneity *p* Value (*I*^2^)
Blood loss					
IBL (mL)	4	171/165	−32.72 [−129.17, 63.72]	0.51	<0.00001 (97%)
HBL (mL)	3	136/130	−76.73 [−178.30, 24.84]	0.14	<0.00001 (94%)
TBL (mL)	3	179/179	−69.65 [−149.93, 10.64]	0.09	0.03 (73%)
HCT (%)					
Preoperative HCT (%)	2	106/100	0.51 [−0.50, 1.51]	0.32	0.32 (1%)
Postoperative HCT (%)	2	106/100	1.87 [−0.48, 4.22]	0.12	0.01 (85%)
PT (seconds)					
Preoperative PT (seconds)	4	124/118	−0.39 [−0.63, −0.15]	0.002	0.21 (34%)
Postoperative PT (seconds)	5	144/138	−0.21 [−0.54, 0.12]	0.22	0.06 (57%)
Hb level (g/L)	2	70/64	−0.74 [−4.67, 3.19]	0.71	0.26 (23%)
Preoperative Hb level (g/L)					
Postoperative Hb level (g/L)	3	100/94	1.84 [−3.38, 7.06]	0.49	0.06 (65%)
FIB (g/L)					
Preoperative FIB (g/L)	3	99/93	−0.10 [−0.32, 0.12]	0.37	0.02 (73%)
Postoperative FIB (g/L)	3	99/93	0.02 [−0.10, 0.14]	0.72	0.90 (0%)
APTT (seconds)					
Preoperative APTT (seconds)	3	79/79	1.12 [0.57, 1.68]	<0.0001	0.93 (0%)
Postoperative APTT (seconds)	3	79/79	−0.30 [−2.47, 1.87]	0.79	<0.0001 (91%)
Drainage volume	4	120/114	−5.97 [−19.04, 7.10]	0.37	0.44 (0%)
Blood transfusion rate	4	140/134	0.91 [0.60, 1.40]	0.68	0.95 (0%)

IBL, intraoperative blood loss; HBL, hidden blood loss; TBL, total blood loss; HCT, hematocrit; PT, prothrombin time; Hb, hemoglobin; FIB, fibrinogen; APTT, activated partial thromboplastin time; I, intravenous; T, topical; RR, risk ratio; MD, mean difference.
